# Examining the Use of Virtual Reality to Support Mindfulness Skills Practice in Mood and Anxiety Disorders: Mixed Methods Study

**DOI:** 10.2196/45640

**Published:** 2024-12-06

**Authors:** Rebecca Blackmore, Claudia Giles, Hailey Tremain, Ryan Kelly, Fiona Foley, Kathryn Fletcher, Maja Nedeljkovic, Greg Wadley, Elizabeth Seabrook, Neil Thomas

**Affiliations:** 1 Centre for Mental Health and Brain Sciences Swinburne University of Technology Hawthorn, VIC Australia; 2 School of Computing Technologies RMIT University Melbourne Australia; 3 School of Computing and Information Systems University of Melbourne Melbourne Australia

**Keywords:** virtual reality, mindfulness, mood disorders, anxiety disorders, depression, bipolar disorder

## Abstract

**Background:**

Virtual reality (VR) has been proposed as a technology to support mindfulness practice through promoting increased engagement and presence. The proposed benefits of this technology have been largely unexamined with clinical populations. Further research is required to understand its clinical potential and utility in improving and managing mental health symptoms.

**Objective:**

This study aims to investigate the proximal impacts of a single, brief, VR-supported mindfulness practice for individuals with a mood or anxiety disorder and to understand user experiences, which may affect the acceptability and efficacy of VR mindfulness for this population.

**Methods:**

This mixed methods study recruited 28 participants with a primary diagnosis of major depressive disorder, bipolar disorder, or anxiety disorder. Participants completed a mindfulness practice wearing a VR headset that was presenting an omnidirectional video of a forest scene, which was overlaid with a guided audio voiceover. Before and after the practice, measures were completed assessing state mindfulness (Toronto Mindfulness Scale), affect (Positive and Negative Affect Schedule), and anxiety (State-Trait Anxiety Inventory Y-1; n=27). Semistructured interviews were then held inquiring about the user experience and were analyzed using thematic analysis (n=24).

**Results:**

After completing the VR-supported mindfulness practice, both measures of state mindfulness on the Toronto Mindfulness Scale, mean curiosity and decentering, increased significantly (Cohen *d*=1.3 and 1.51, respectively; *P*<.001). Negative affect on the Positive and Negative Affect Schedule (Cohen *d*=0.62; *P*=.003) and State-Trait Anxiety Inventory Y-1 state anxiety (Cohen *d*=0.84; *P*<.001) significantly reduced. There was no significant change in positive affect (Cohen *d*=0.29; *P*=.08). Qualitative analysis of interviews identified 14 themes across 5 primary theme categories. The results suggested that being mindful during the use of the app was experienced as relatively effortless because of the visual and immersive elements. It was also experienced as convenient and safe, including when compared with prior traditional experiences of mindfulness. Participants also identified the uses for VR-supported mindfulness in managing emotions and symptoms of mental illness.

**Conclusions:**

The results provide preliminary evidence that VR-supported mindfulness can improve emotional states and manage mental health symptoms for those with mood or anxiety disorders. It offers some potential clinical applications for those with mood or anxiety disorders for exploration within future research.

## Introduction

### Background

Mindfulness has been described as a purposeful quality of attention toward the present moment involving an attitude of nonjudgment and curiosity [1,2]. Mindfulness-based approaches have increasingly been used as psychological interventions, with demonstrated benefits for a range of mental health outcomes, including depression, anxiety, and stress [3]. Several mechanisms have been proposed to explain how mindfulness skills lead to symptom reduction and behavior change. Although conceptualizations vary, mindfulness practice has been posited to affect change via modifying emotion regulation (reappraisal of emotions, exposure, and extinction), attention regulation, body awareness, and perspective of the self [4,5].

Mindfulness-based approaches are promising interventions for clinical populations. Mindfulness-based approaches can reduce depressive and anxiety symptoms both in primary anxiety and depressive disorders [6] and other mental health conditions, such as bipolar disorder (BD) [7,8]. The mechanisms through which mindfulness operates may be of particular relevance for clinical populations. For example, a qualitative metasynthesis of clinical studies [9] found that following mindfulness, participants reported observing and responding to early warning signs more quickly, acknowledging and accepting their extreme emotional states, which may prevent spiraling: observing fewer negative impacts of episodes (such as shame) and improved perspectives of themselves (eg, as distinct from their symptoms and challenges).

Psychological mindfulness-based interventions (MBIs), such as Mindfulness-Based Stress Reduction [2] and Mindfulness-Based Cognitive Therapy [10], primarily focus on learning mindfulness via regular practice in meditation. However, some studies suggest that learning mindfulness via traditional meditations may be demanding and aversive, and this may be particularly salient for clinical populations [11,12]. A metasynthesis of qualitative studies of group MBIs in clinical populations identified challenges, including being confronted with one’s difficulties with fresh awareness, and, in some cases, experiencing more distress than before commencing, and practical concerns, such as finding the time and physical space to practice and difficulty grasping the core concepts of mindfulness [9]. Difficulties with maintaining stillness and concentration have also been reported, especially in individuals with BD [13].

Although traditionally being learned via formal meditation practices, mindfulness may be cultivated using any number of techniques and short exercises that support attention orientation to the present moment and acceptance [1,14]. This more flexible definition of mindfulness coincides with an increasing use of brief MBIs as alternative ways of developing mindfulness skills [15]. Brief MBIs, including one-off and stand-alone practices of as little as 5 minutes, have demonstrated benefits for outcomes, including depression, anxiety, negative affect and mindfulness, as well as altered neural activity [4,15,16]. Briefer, more accessible mindfulness practices may therefore provide the benefits of mindfulness while addressing challenges associated with other practices.

Digital technologies, such as virtual reality (VR) apps, can support mindfulness practice. Interactive and immersive technologies, such as VR apps, may support components of mindfulness, such as emotion regulation and attention [17]. VR technology typically encompasses a 360° head-mounted display, with hand controllers or natural gestures that enable the user to interact with a computer-generated virtual environment (VE). VR apps can be created using either computer-generated imagery [18] or omnidirectional video footage [19].

The features of VR may provide accessible mindfulness practices, which address some of the barriers associated with mindfulness. VEs provide the user with the sensation of being located within the environment, contributing to the psychological sense of presence or “being there” [20]. VEs are highly engaging, immersive, and interactive, which may facilitate concentration during mindfulness practice [21,22]. Furthermore, access to natural environments during mindfulness practice has been found to offset the attentional effort of developing mindfulness skills [23] and increase adherence [24]. These potential benefits may be of particular relevance for individuals with anxiety and mood disorders, for whom distractibility and difficulties sustaining attention and concentration are hallmarks [25]. Participants are free to choose their own anchors and interaction with the environment, allowing a personalized experience [21] and potentially minimizing the aversion associated with some prior practices. Indeed, compared with non–VR mindfulness, reported benefits have included improved adherence [26,27] and mindfulness [27].

Several studies have piloted the use of VR for mindfulness skills practice in nonclinical populations, showing promising findings with regard to improving state mindfulness and positive affect as well as general acceptability of the apps [21,22,28]. For example, a mixed methods study by Seabrook et al [21] studied the utility of VR for supporting mindfulness practice using omnidirectional video of a natural forest environment, which was overlaid with a guided audio voiceover. Nonclinical participants reported feelings of peace, a sense of presence in the VE, having present moment awareness, and being able to use the natural VE to direct or control attention.

Several pilot studies have investigated VR for mindfulness skills practice for mental health conditions, such as depression and anxiety disorders [26,29,30]. Navarro-Haro et al [26] found that MBI groups with and without VR mindfulness skills training showed similar improvements in symptoms of anxiety, depression, and emotion regulation, but the VR group was more adherent to the program. Tarrant et al [30] compared a brief, nature-based mindfulness experience to a resting control group with individuals with self-reported anxiety symptoms. The results showed that both the VR experience and the quiet rest control condition reduced anxiety. However, the VR experience was very brief, 5 minutes in total, and no measure of state mindfulness was administered; therefore, it was unclear how either of the conditions contributed to mindful attention. Further research is required to understand potential clinical applications of VR technology and whether it has the capability to improve the ease of mindfulness practice, support mindful attention, improve affect, or reduce symptoms of anxiety among those with mental health conditions [31]. It is also important to explore the suitability and safety of this technology, particularly in its application to clinical groups and individuals experiencing mental health disorders. The use of novel immersive technology for mental health interventions may carry specific risks that are yet not well understood. Thus, the safety of using VR for mindfulness practice with mental health disorders should be evaluated through pilot studies before long-term use in large trials.

### This Study

Using a mixed methods design, this study aimed to investigate the proximal effects of a single-session, VR-supported mindfulness practice [21] on state mindfulness, affect, and state anxiety with a sample of individuals with mood or anxiety disorders. This study also explored the potential uses and clinical suitability of VR-supported mindfulness as a mental health self-management tool. Qualitative assessment explored the participants’ experience of completing the mindfulness practice, comparison with their previous experiences of mindfulness practice, and the potential uses participants envisaged for VR-supported mindfulness as a tool in supporting their mental health self-management.

## Methods

### Ethical Considerations

All procedures received ethics approval from the Swinburne University of Technology Human Research Ethics Committee (SHR 2018/256) and the University of Melbourne Human Research Ethics Committee (ID# 1852613.2). All participants gave full informed consent, data were deidentified, and participants received AUD $80 (USD $50) reimbursement for their participation.

### Participants

Participants were recruited via promotion on Facebook and emails to mental health websites and services. Interested participants completed a web-based screening survey assessing their eligibility. Inclusion criteria were aged 18 to 65 years; fluent in the English language; reported normal or corrected-to-normal vision and hearing; diagnosis of BD, major depressive disorder (MDD), or anxiety disorder; and willingness to engage in a mindfulness session. Participants were excluded if they reported a diagnosis of photosensitive epilepsy; had previous experience of severe cybersickness; or were currently experiencing a mood episode, active suicidality, or psychosis. Eligibility was confirmed by telephone using the Mini International Neuropsychiatric Interview [32] and the Columbia-Suicide Severity Rating Scale [33]. Of 55 participants assessed for eligibility, 34 met the criteria, and a final sample of 28 took part (4 did not respond to further contact and 2 were canceled because of the COVID-19 pandemic), of which 24 additionally took part in the qualitative interviews.

### Measures

#### Baseline Variables

To characterize the sample, a demographic questionnaire asked participants to identify information related to their sex, age, education level, history of mental illness, mental health treatment, and use of prescribed medications. Participants were also asked about their previous experience with mindfulness and confidence and experience in using VR technology.

#### Five Facet Mindfulness Questionnaire Short Form

The Five Facet Mindfulness Questionnaire Short Form (FFMQ-SF) [34] total score was used to assess baseline trait mindfulness. The FFMQ-SF asks respondents to rate statements, indicating their general tendency to be mindful in daily life. Statements are rated on a response scale from 1=never or very rarely true to 5=very often or always true. The measure shows good internal consistency and convergent validity with the full version of the questionnaire [35]. In the current sample, Cronbach α scores ranged between 0.62 and 0.83 for the 5 scales, which indicated adequate reliability.

#### Pre-Post Intervention Measures

##### Toronto Mindfulness Scale

The Toronto Mindfulness Scale (TMS) consists of 13 items asking participants to consider the degree to which they were mindful in the previous 15 minutes [36]. The measure consists of 2 distinct subscales, curiosity (6 items) and decentering (7 items). Response options are rated on a 5-point Likert scale ranging from 1 (not at all) to 5 (very much). Higher scores indicate greater association with each construct. A strong internal consistency of 0.88 for curiosity and 0.84 for decentering has been previously reported [36]. This study found an acceptable Cronbach α of 0.88 and 0.90 for curiosity before and after viewing the VR-supported mindfulness practice, respectively. The results for the decentering scale were acceptable (α=0.68 before and α=0.72 after the practice).

##### Positive and Negative Affect Schedule

The Positive and Negative Affect Schedule (PANAS) [37] was used to measure state positive and negative affect. This is a self-report measure comprising two 10-item scales measuring positive and negative affect. Items are rated from 1 (*very slightly or not at all*) to 5 (*extremely*), producing positive and negative affect scores ranging from 10 to 50. Cronbach α for the positive affect scale was 0.89 (before) and 0.90 (after), and the negative affects scale was 0.88 (before) and 0.72 (after).

##### State-Trait Anxiety Inventory Y-1

The State-Trait Anxiety Inventory Y-1 [38] was used to measure state anxiety**.** This self-report measure of trait and state anxiety includes 20 state anxiety items that were used in this study. Response options are rated on a 4-point scale. Higher scores indicate higher self-perceived state anxiety. Normative adult sample data have shown strong internal consistency coefficients ranging from 0.86 to 0.95 [38,39]. In this study, the resultant Cronbach α of 0.93 both before and after the VR-supported mindfulness practice indicated good internal consistency.

#### Semistructured Interviews

A semistructured interview guide was developed for this study. Questions were arranged into topics, including feedback and comments on the VR experience, the use of mindfulness in VR for participants both with and without prior mindfulness experience, potential impact of mental health symptoms on use of the program, and potential uses for the app. A copy of the interview guide is available from the authors on request.

### VR Mindfulness App

The app, called Place, was used to deliver a 15-minute program of guided mindfulness within a VE [21,40]. The app was developed through a user-centered design process and has been evaluated with a general population sample [19]. Crucially, the app has been shown to create positive changes in mindful awareness as well as being safe and not producing any related simulator sickness. The VE consisted of 2 forest scenes created from omnidirectional footage: first, a clearing next to a river and, second, at the edge of a river with a waterfall in view. The audio guidance used was tailored to the VE and invited the user to attend to physical sensations, the breath, different parts of the VE, and prompting the user to redirect their attention if they notice their mind wandering. The voiceover also allowed space for unguided practice for up to 70 seconds at a time. A recent article articulated some of the challenges in designing and evaluating digital interventions for mental health, providing a framework for classifying such interventions [41]. Within this framework, the Place app was designed for *experiential* (as opposed to *didactic*) learning and as an *on-the-spot* (as opposed to *offline training*) tool; that is, it is intended that users practice mindfulness while using the app and that, ultimately, the app is used when and as needed within users’ real contexts. Although there is evidence that even brief mindfulness practices may lead to altered neural activity and longer-term or “downstream” improvements [4,15,16,42] (within this framework classified as *offline training*), this is not the focus of the present investigation.

### Procedure

After completing a telephone assessment, participants attended an in-person session in research rooms in a university setting. Participants completed the questionnaires electronically via the survey software Qualtrics, followed by the 15-minute VR mindfulness practice completed with an Oculus Go headset, with 2 additional minutes of instructions and safety information provided verbally by research staff. Participants were seated in a soundproof booth with headphones and a swivel chair, which allowed for exploration of the 360° environment. Participants then completed quantitative postexercise measures, followed by the optional qualitative semistructured interviews. Interviews were audio recorded and transcribed by NVivo. Transcripts were checked, and any errors were corrected. Interviews were completed by 4 postgraduate-trained research assistants, who received training from the investigators, which included practice interviews.

### Data Analysis

Descriptive statistics were processed and analyzed in IBM SPSS Statistics (version 26.0). Normality and skewness checks were conducted, and Levene test for equality of variances showed no violations of assumptions. Paired sample *t* tests (2-tailed) were conducted to compare pre- and posttreatment scores across measures as the primary analysis, with repeated measures ANOVAs with a Cronbach α level of 0.05 conducted to investigate differences between clinical groups across measures and timepoints.

The qualitative data were analyzed using an inductive approach to thematic analysis [43]. Transcripts were open coded to attribute nominal codes. A secondary coder was allocated 3 transcripts at random to code, and 1 theme was altered as a result. As a participant checking procedure, all participants who took part in the interviews were emailed the initial themes and invited to provide feedback: 4 participants opted to provide feedback, all of whom endorsed the themes.

## Results

### Overview

Of the 28 participants recruited for the study, 1 was excluded because of an IT issue impacting their time 2 data. As a result, 27 participants were included in the final analysis: 17 female participants and 10 male participants. The participants had a mean age of 40 (SD 11.15; range 20-63) years. All participants endorsed prior experience of counseling or psychotherapy, with 15 (56%) participants receiving therapy in the past month. In total, 20 (74%) participants indicated they were currently taking medication as part of their current treatment. Table 1 presents the demographic characteristics of the sample (n=27).

Primary diagnoses included MDD (n=9), BD (n=10), and anxiety disorders (n=8), which included generalized anxiety, social anxiety, and panic disorder. A total of 14 participants had secondary diagnoses: 8 (57%) of which were anxiety disorders and 6 (43%) were MDD. We noted no statistically significant baseline differences between clinical groups (MDD, BD, and anxiety) on trait mindfulness (FFMQ-SF total score: *F*_2,24_=0.25, *P*=.78) or any of the measures used in the pre-post analyses (TMS curiosity: *F*_2,24_=0.48, *P*=.63; TMS decentering *F*_2,24_=0.30, *P*=.74; PANAS positive affect *F*_2,24_=0.20, *P*=.82; PANAS negative affect *F*_2,24_=0.39, *P*=.68; State-Trait Anxiety Inventory Y-1: *F*_2,24_=0.15, *P*=.86).

Most participants (24/27, 89%) had experience of mindfulness practice, although 3 (11%) reported not having tried it before but knew of it. Most participants (20/27, 74%) reported feeling very to extremely confident in using technology, and 63% (17/27) of participants had previous experience with VR. The mean total score of the FFMQ-SF was 44.3 (SD 7.6).

**Table 1:** Demographic characteristics of the sample (n=27).

Sample characteristics			Values, n (%)
**Education**			
	Completed secondary school	1 (4)	
	Some tertiary education	7 (26)	
	Completed tertiary education	8 (30)	
	Some postgraduate education	3 (11)	
	Completed postgraduate education	8 (30)	
**Relationship status**			
	Single	9 (33)	
	Partnered	4 (15)	
	De facto	3 (11)	
	Married	6 (22)	
	Separated	2 (7)	
	Divorced	3 (11)	
**Primary diagnosis**			
	Bipolar disorder	10 (37)	
	Major depressive disorder	9 (33)	
	Generalized anxiety disorder	5 (19)	
	Social anxiety disorder	2 (7)	
	Panic disorder	1 (4)	
**Previous mindfulness experience**			
	None, but have heard of it	3 (11)	
	Tried it once	3 (11)	
	Tried it a few times (2-5)	13 (48)	
	Do it monthly	1 (4)	
	Do it weekly	4 (15)	
	Do it daily	3 (11)	
**Previous** **virtual reality** **experience**			
	None, but have heard of it	10 (37)	
	Tried it once	8 (30)	
	Tried it a few times (2-5)	8 (30)	
	Do it monthly	1 (4)	
**Confidence with technology**			
	Slightly	1 (4)	
	Moderately	6 (22)	
	Very	13 (48)	
	Extremely	7 (26)	

### Pre- and Postintervention Measures

The mean effort participants rated it took to be mindful in the program was 37.6 out of 100 (SD 26.7), with 70% (19/27) of participants rating this less than 50. Table 2 presents changes in measures from before to after experiencing the VR-supported mindfulness practice. Scores on both subscales of the TMS increased significantly (*P*<.001), with large effect sizes. Both state negative affect and state anxiety reduced, also with large effect sizes. There was no change in state positive affect. Repeated measures ANOVAs indicated no main effect of diagnostic group (MDD, BD, and anxiety) or time×group interaction, and similarly, no interactions were observed for recent receipt of therapy or whether taking medication.

**Table 2:** Mean scores on pre- and postintervention measures (n=27).

Measures	Preintervention scores, mean (SD)	Postintervention scores, mean (SD)	*t* test (*df*)	*P* value	Cohen *d*
TMS^a^ curiosity	15.81 (5.76)	22.03 (5.04)	6.93 (26)	<.001	1.30
TMS decentering	16.93 (4.53)	24.00 (4.48)	8.07 (26)	<.001	1.51
PANAS^b^ positive affect	28.26 (6.65)	30.30 (7.03)	1.83 (26)	.08	0.29
PANAS negative affect	14.90 (4.83)	11.56 (2.04)	3.33 (26)	.003	0.62
State-Trait Anxiety Scale Short Form state anxiety	41.96 (9.70)	32.41 (8.42)	4.49 (26)	<.001	0.83

^a^TMS: Trait Mindfulness Scale.

^b^PANAS: Positive and Negative Affect Scale.

### Semistructured Interviews

#### Overview

In total, 24 of the participants agreed to participate in a semistructured interview to gain a better understanding of the user experience of the VR-supported mindfulness practice with a clinical population. A total of 14 themes were developed across 5 primary theme categories: ease of present moment awareness, multilayered engagement in mindfulness, comparisons to practicing mindfulness outdoors or in public settings, helping develop mindfulness practice, and supporting and interacting with mental health (Table 3).

Within many of the identified themes, participants reflected on their experiences with the Place app in comparison to their previous mindfulness experiences, which may illuminate the *unique* benefits and challenges of VR mindfulness. Where such comparative statements contributed the theme (in contrast to highlighting characteristics or experiences of the intervention more broadly), these have been identified within their relative themes.

**Table 3:** Qualitative results.

Theme category and themes		Illustrative quotes
**Ease of present moment awareness**		
	Reduced external distractions	“I liked that. It was focused so that I wasn't distracted by surrounding noises because I didn’t know what was happening in the real world or real environment*.*” [P24]
	Positive emotions are expected and experienced	“...my meditation videos...has to be about an hour that I feel like I'm like, relax...but this one [the app] like in 15 minutes, I was very calm” [P9]
	Embedding parts of regular mindfulness practice	“...when I was watching the water, I was trying it out and saying if you watch the river move, then that’s kind of the same thing as radical acceptance because you can only accept what’s going on in the second that it’s happening*.*” [P9]
**A multilayered engagement in mindfulness**		
	Being able to see things adds to feeling involved	“...I've done a lot of auditory [mindfulness practice]...I was unaware how much I actually really need both things [auditory and visual] to be stimulated for actually get me really in a calm, mindful place” [P9]
	Not focusing on internal elements was both helpful and confusing	“I get frustrated with mindfulness things where it’s all about breathing...I just get bored with that. So I liked having something else to focus on...” [P16]
**Comparisons to practicing mindfulness outdoors or in public settings**		
	A convenient way to access mindfulness in nature, indoors	“...regardless of...your mood or your anxiety...just chuck it on...and you can immediately start work on the mindfulness side of things without the 10 hour round trip to do it.” [P9]
	A sense of safety in the virtual environment	“just more awareness of what’s around you that’s safe and guided. Because often I try to practice mindfulness outside in different environments that I may not have control over...but making sure that this has been overseen and this is a practice thing. Just made me feel a lot more safe” [P1]
**Helping to develop mindfulness practice**		
	Easing into different forms of mindfulness practice	“[the app] helps to engage you in the moment, which I think is probably one of the hardest things about mindfulness. And once you’ve gotten to that point, then you can take that next step to reflecting and exploring.” [P7]
	Encouragement to start mindfulness again	“I think that I’d get the benefits of it, which would mean that I’d probably do it more often...” [P24]
**Supporting mental health**		
	Use for increasing present moment awareness	“...when I’m depressed, I feel like I’m just dissociating and losing time. So doing that [the app] sort of brings me back to the present mind.” [P17]
	Changing emotions and getting away	“If there was some kind of a trigger that had happened, if I’d had an argument, whatever the trigger was, if I was feeling that way, I would probably gravitate towards it [the app] in order to feel calm, and then be able to investigate and associate the emotions and feelings that I had...” [P23]
	Disorder-specific uses	“I think it could be a way of again, kind of stepping back...stepping down some of the intensity of feeling [when mood is elevated] and just some moments of calm in the rush...” [P16]
	Use for prevention	“...depends if you notice you’re in an elevated mood state because often you don’t or don’t until it’s too late. So if you catch it in time really early, then yes, I think this would be helpful...” [P9]
	Barriers to using virtual reality during a mental health episode	“...one of the associations with depression is you don’t feel like doing anything so...I: So that might be something that would get in the way...” [P2]

#### Theme Category 1: Ease of Present Moment Awareness

Participants reported that being mindful, or having present moment awareness, while using the app was relatively easy.

##### Reduced External Distractions

External distractions in previous mindfulness practice were noted, in contrast to a sense of reduced distractions while using the app:

You need that personal, alone [time], which is really hard to get in life...It’s a bit hard to lock yourself in a room and have no noise...whereas this [the app] just allows that.

P24

Within this theme, participants drew comparisons between experiences with the app and previous mindfulness experiences. The headset was a contributor to accessing this space:

You put the headset on...And it makes it easier. The extra sensory stimulation lends itself to the sense of being in a “bubble.”

P24

I feel like [the app is] a more all-encompassing sensory experience...And I felt like if you do a guided meditation at home, you're very aware you're at home in your lounge room. But this was...I feel like I was in a bubble...It really helped a lot.

However, one participant found the images in the VE distracting in comparison to previous mindfulness experiences.

Like it’d take an effort for me, a bit more than usual, to do the mindfulness exercise [using the app] because of the fact that I was distracted by the images.

P15

##### Positive Emotions Are Expected and Experienced

Positive emotional experiences were accessed sooner using the app, such as feeling peaceful:

...I didn’t achieve that sense of peace until I’d been practicing an hour a day, or more calm and relaxed.

P20

Participants also drew comparisons with prior experiences of mindfulness within this theme. For participant 20, practicing mindfulness with VR contrasted to their prior experiences of mindfulness in a Mindfulness-Based Stress Reduction course, where there had been a period of emotional discomfort:

...[the app] kind of skipped over the part where you’re sitting there going ok I’m supposed to just be focusing on my breath... [and that causes] it’s not even anxiety, but aggravation?

##### Embedding Parts of Regular Mindfulness Practice

Participants integrated their existing mindfulness practice to enhance the experience of the app:

Looking at the trees and imagining their roots growing down into the soil and thinking of that as a grounding kind of exercise...I thought that was a bit you could run with and think about for an extended period of time.

P16

#### Theme Category 2: A Multilayered Engagement in Mindfulness

A perceived importance of having multiple sources of sensory input in practicing mindfulness was further enhanced by the VR app.

##### Being Able to See Things Adds to Feeling Involved

Again within this theme, participants contrasted their experience with other mindfulness practices. For example, the visual element of the app was important in providing a level of stimulation and engagement compared to previous audio-only mindfulness practices:

I think it really draws you in because it’s visual and it is 360°...so it lands you quicker in the experience rather than if you’re just imagining it in your head.

P19

For those participants who had previously practiced mindfulness involving guided imagery, such as imagining leaves floating down a stream, the app provided the image so that there was less attentional fatigue:

I can visualise things really well, but it requires quite a lot of effort...So with this [the app], having the visual there was quite relaxing.

P18

Participants identified that they may gravitate toward different mindfulness practices when they are in different states. The visual aspect of the app may be more useful for calming down rather than a more introspective mindfulness practice:

they feel different [the app and audio guided mindfulness] and I think if I was wanting to feel more calm, I would automatically go for the VR version. If I was in a place where I was willing to be more reflective, I personally would find it easier to do it with audio only.

P7

##### Not Focusing on Internal Elements Was Both Helpful and Confusing

This theme also involved comparisons to other mindfulness modalities. For some participants, the focus on the VE resulted in a reduced focus on internal elements such as the breath, emotions, and thoughts. This was perceived as helpful in staying in the present moment*.* Participant 7 found the visual focus improved present moment awareness:

I didn’t have sort of that enquiring or curiosity...about the thoughts that I was having [in the app]...I have done other audio mindfulness in the past. I think the difference being that when you have something visual to take up your attention, it’s quite in-depth and you’ve got a lot of focus on...it kept me present better than when I’ve done it with just audio.

Other participants indicated that focusing on external stimuli created confusion in whether their focus should be on the self, the environment, or both:

when he was talking about the breathing...I was closing my eyes and then I thought oh but if I’m closing my eyes then I can't see the forest. So that was a bit confusing as to what I should do.

P22

#### Theme Category 3: Comparisons to Practicing Mindfulness Outdoors or in Public Settings

The app was viewed as a convenient and safe way to practice mindfulness in public settings.

##### A Convenient Way to Access Mindfulness in Nature: Indoors

Participants described the app as convenient and an alternative to practicing outdoors. The app was not seen as a replacement but could be used when outdoor spaces were unavailable.

##### A Sense of Safety in the VE

Participants felt safe using the VE and again drew on relative experiences within other modalities within this theme, “even though it’s virtual reality, sometimes it’s like that is safer than the real world,” and this was related to “...the real world [being] very confronting, people especially...” (P8).

There was a sense the VE was more predictable and a trust that nothing threatening would appear as part of the experience.

#### Theme Category 4: Helping to Develop Mindfulness Practice

This theme identifies some ways the VR app could be used both for initiating and restarting regular mindfulness, encompassing a longer-range view that the app may be used to develop mindfulness skills over time.

##### Easing Into Different Forms of Mindfulness Practice

The app could benefit beginners to mindfulness practice. Participant 16 noted this was important for those with *histories of trauma*:

...[because] it’s really hard to get in touch with our bodies...[and] having a way...to maybe ease into it [mindfulness practice] through an external pathway could be really effective.

##### Encouragement to Start Mindfulness Again

The app prompted a reconsideration to initiate a regular mindfulness practice for those participants who had been unsuccessful or found barriers. Participants stated they would be encouraged to practice more regularly.

#### Theme Category 5: Supporting Mental Health

Participants described the app as providing short-term “outcomes,” such as improving state mindfulness, mood, and mental health symptoms.

##### Use for Increasing Present Moment Awareness

The app was used to achieve a state of mindfulness or a way of directing attention back to the present.

##### Changing Emotions and Getting Away

The VR app could be used to reduce heightened emotions during a period of ill mental health. Participants described the notion of “getting away” as the desire to take a break from these emotions or stressors:

It would help with trying to slow down some of those thoughts that come with the depressive episode.

P7

Some participants reported using the program to “let go” or sort through thoughts that were bothering them:

So part of it is negative thoughts that you can’t quite get away from. Having mindfulness in general and the VR, as part of that, to let you get into a space where you're letting go of those feelings, those thoughts. That’s where it could be very helpful.

P2

Related to this was the notion of a “reset,” where participants used the program to take a break before continuing a busy or stressful day:

So it would be something that I would like to use, even if it’s a 5 or 10 minute exercise in the middle of a crazy day, just to regroup and re-energize.

P19

##### Disorder-Specific Uses

The app could improve motivation or energy:

With depression you can often feel like really flat and lethargic and lack of motivation. So I think that [the app] can help with feeling energised too.

P11

The potential for this to be translated into taking action and “doing something” was noted:

Whether it’s sparking my interest in my environment or just distracting myself from my mood or wanting to go out and do something afterwards, I think that would be a good exercise to improve my mood.

P1

For participants with an anxiety disorder, the program was viewed as a way of reducing anxiety before stressful activities:

Knowing the shift I felt in fifteen minutes from the beginning to the end...if I had something that I was anxious about doing I would totally do that first...And then go do...the activity...

P20

Participants with a diagnosis of BD discussed using the program to manage elevated mood or as a way of “stepping back” from this.

##### Use for Prevention

The program may be useful as a preventive mental health strategy. With regard to depressed mood:

Ironically, I would probably use it more in that early part...because I know that it helps...and at that point in the early part of a depressive episode, I do want to help myself and I think I would reach for something like that.

P7

Participant 15 spoke about breaking the cycle for worrying:

...probably being especially for people who get that circular thinking, being a bit of a circuit breaker before we kind of spiral.

Similar to the outcome focused uses, such uses were deemed as reacting to the signs of developing mental health symptoms in the hope to stop it at that moment rather than developing skills over time.

#### Barriers to Using VR During a Mental Health Episode

During a period of ill mental health, there may be barriers, such as a lack of awareness or insight, which may limit the usefulness of the app:

I just know me. I don’t think I would. And I feel like a different person in that state [manic or hypomanic]. And I don’t do really anything that’s a good decision at that point so, I would find it unlikely.

P7

For participants with a diagnosis of MDD, low motivation to engage in daily activities could prevent use of the app:

Yeah, but that that wouldn’t be an objection to the to the VR…

P2

## Discussion

### Principal Findings

This study aimed to investigate the effects of a single, brief, VR-supported mindfulness practice on individuals with a mood or anxiety disorder and well as to explore the potential uses of this technology as a mental health management tool. The results from this study suggest that for individuals with a mood or anxiety disorder, VR apps, such as Place, have potential to improve symptoms in the short term as well as offering a safe and acceptable tool for the self-management of mental health symptoms.

Baseline FFMQ scores reflect levels of trait mindfulness, and our FFMQ mean scores were slightly lower than average scores found in the general population [21,44]. Following the VR-supported mindfulness experience, there was a significant increase in state mindfulness, suggesting that the VR app was successful in enhancing and supporting mindfulness practice. This finding is consistent with other research in the field [22,26] as well as a previous study, which investigated the use of this VR app with a general population sample [21].

There was a significant reduction in state anxiety following the VR-supported mindfulness experience. These results contribute to a growing body of evidence for the use of VR in improving anxiety symptoms [30,45,46]. There was no significant change in positive affect following the VR experience; however, there was a significant reduction in participants’ negative affect. These results differ to those of Seabrook et al [21] who found that positive affect increased, but there was no change to negative affect following the VR experience in a nonclinical population. Furthermore, there have been associations between mindfulness and positive affect found in previous studies [47]. Seabrook et al [21] used single-item measures of positive and negative affect, and it is not possible to directly compare results to this study. Nonetheless, there was a trend toward increased positive affect after the intervention, and it is likely there was insufficient power to detect small magnitude changes in this study. Furthermore, while this study had a clinical sample, positive affect scores were comparable to the general population (while negative affect scores reflected those from clinical samples), potentially resulting in a ceiling effect for positive affect [48]. The aim of mindfulness practice is not to directly alter mood states, yet mindfulness targets emotion regulation [49]. Therefore, the benefits of mindfulness for positive affect may be more evident for those experiencing low positive affect or affect variability (ie, dysregulation) than participants in this study. Further research with larger samples and time series data is needed to elucidate these relationships.

A plausible alternative explanation for the changes participants reported to anxiety and negative affect following engagement with the app is that participants were engaging in distraction (disengagement from their present moment experiences), rather than mindfulness. For example, 1 participant observed that the app allows “you [to] have something visual to take up your attention, it’s quite in-depth and you’ve got a lot of focus on...it kept me present better than when I’ve done it with just audio.” However, state mindfulness improved significantly after the intervention, suggesting that, at the group level, mindfulness was effectively targeted. This aligned with participants’ self-assessments of having experienced mindfulness while using the app. Furthermore, prior research has suggested that more immersive, multisensory mindfulness practices and connectedness to nature both confer benefits, including increased mindfulness [27,50]. Nonetheless, it is possible that some individuals used the VE for mindfulness and others for distraction. Furthermore, central to mindfulness is noticing distraction and bringing attention back to the present. It is also possible that reducing external distractors with the VE inhibited this central aspect of mindfulness. Therefore, the potential role of distraction in the observed benefits of the VR mindfulness warrants further investigation.

Our key qualitative findings were that participants found being mindful during the VR app less effortful than previous experiences of mindfulness, specifically around attentional effort. Participants reported integrating their existing mindfulness practice skills to the app to enhance the experience. In this study, participants made direct contrasts to previous experiences, including assisting with maintaining attention, improving participants’ ability to focus on their present moment experience and immersion in the practice, inducing a state of calm and reducing discomfort previously associated with mindfulness, and increasing sense of safety relative to practicing mindfulness outdoors in real-world environments. External distractions were less noticeable because of the multisensory experience of the app, leading to the feeling of being in “a bubble.” Given the barriers some clinical populations face in commencing formal mindfulness practice, the app contributes to an ease of present moment awareness because of both the high level of presence offered by VEs [31] and the possible benefits of viewing a natural environment in reducing attentional demands [23,51].

Among the novel findings within this study were those relating to the utility of the app for clinical populations. This study lends support to VR mindfulness as a safe and acceptable tool for those with mood and anxiety disorders, with the potential to improve negative affect and anxiety. This finding adds to the limited, yet growing, body of literature investigating VR mindfulness in clinical populations [26,29,30]. In addition, the findings suggest that there may be unique benefits associated with VR mindfulness for different clinical groups. Specifically, participants with depression highlighted that the app may help improve motivation, interest, and energy as well as reducing the likelihood of dissociation. Those with anxiety disorders speculated that it may help them reduce their anxiety in preparation for a stressful event. Those experiencing BD identified the app as having potential to help them manage elevated moods by stepping back. These findings are speculative yet suggest that there may be unique benefits of VR mindfulness for different clinical groups, providing novel directions to be further explored within future research.

Conversely, some participants identified that their mental health symptoms may interfere with their use of the app, with the perception that they are less likely to use, or benefit from, the app when experiencing more severe symptoms. In line with this, the severity of mental health symptoms has been identified as a common barrier for accessing technology-supported mental health interventions [52]. Therefore, the role of symptoms in engagement is an essential consideration for the use of VR mindfulness in clinical populations. One possibility that emerged from qualitative findings within this study is the potential for VR mindfulness to be used as a preventive measure, which could provide the additional benefit of circumventing this barrier. Mindfulness as a preventive intervention is not novel [53,54]; however, the potential benefits of VR mindfulness as a preventive tool for clinical populations is, to the authors’ knowledge, yet to be explored.

Focus on the visual stimuli was generally seen as engagement and present moment awareness. However, for 1 participant, it was not seen as compatible with their understanding of prior mindfulness practice in which they closed their eyes. Previous focus group evaluations of the app reported that participants expressed a desire to close their eyes because of attentional fatigue and to match previous mindfulness experiences [40]. Visual stimuli are attentionally favored when both visual and auditory stimuli are presented [55,56]. This may be an important consideration for future development of the audio voiceover used for the app, with directions for participants to open and close their eyes offered as part of the introduction to the exercise.

Participants’ perspectives suggested that the app might be used as a starting point in learning mindfulness skills. It was noted that for participants previously finding it difficult to connect with their bodies, the app enabled a more externally focused practice. While participants may see the value in focusing inward, as is commonly the approach in MBIs, they may not be ready to do this straight away. An approach of focusing out before focusing in may support participants who may otherwise be confronted with an increased awareness of internal state, with this experience of confrontation or awareness of unpleasant internal experiences being echoed in prior qualitative research [9,12].

Participants indicated perceiving multiple potential future uses of the app in supporting their mental health self-management including the goals of altering emotional state and increasing mindful awareness. Some identified benefits were perceived as addressing challenges participants had faced with previous mindfulness practices, and others were characteristics of the present intervention. For example, participants suggested the app could function as both a mindfulness and emotion regulation tool, depending on the goals of their mental health treatment. Our results indicate that the use of this VR app is associated with reduced state anxiety and negative affect, making the present moment more appealing. In a study by Riva et al [57], emotional valence and felt presence (as opposed to present moment awareness) were found to be related in VEs, such that environments with more emotionally prominent features (eg, experimentally manipulated relaxing or anxious environments) evoked higher felt presence than emotionally neutral environments. It is possible that participants who found the environment relaxing in this study were more able to feel involved and engaged in the environment, in turn supporting present moment awareness. In addition to participants reporting a feeling of safety within the VE, there was also a lack of reported adverse events associated with the study.

Given the dearth of literature investigating VR mindfulness in clinical populations, there is currently inconclusive support for benefits of VR mindfulness over and above mindfulness interventions more broadly [58]. This study identified some potential unique benefits of the app relative to other mindfulness practices based on participants’ qualitative experiences. In line with these findings, a recent systematic review found that more immersive VR-based mindfulness training was more effective than either less-immersive VR (eg, 2D computer-generated scenes) or conventional mindfulness training for improving engagement and adherence and for improving mindfulness levels in clinical and nonclinical populations [27]. While participants in this study offered reflections based on their prior experiences with mindfulness, we did not directly compare the VR app with other mindfulness practices. Firm conclusions cannot be drawn based on these data; however, they offer some potentially informative directions warranting further exploration.

Some of the barriers to conventional mindfulness practice may translate to VR mindfulness as well. Participants reported a potential lack of motivation to practice mindfulness during depressive episodes, which was consistent with previous research with individuals with BD [13]. In this study, barriers relating to using the VR app to improve mental health were almost solely identified by those with a diagnosis of MDD or BD, suggesting these groups may be at greater risk of difficulties in engagement compared to those with an anxiety disorder. Given the limited context for using the app this study, it was not possible to corroborate the opposing perspective of some participants that the app may help improve energy and motivation. However, as noted, a recent review found VR mindfulness was associated with better engagement and adherence than non–VR mindfulness [27]. Future research could investigate how to increase the likelihood that participants will engage with the app: this might include enhancing the design of the VR experience. There is potential for integration with other areas of developing digital technologies, such as ecological momentary intervention.

Important limitations of this study were its sample size and context, both limiting the generalizability of findings. A single app was used, using 360° video environments and with audio guidance, so findings may not generalize to other types of VR-supported mindfulness apps. The testing session was a one-off experience in a laboratory setting, and the results may not generalize to real-world environments, where distractions and ease of focus may differ. The study sample was small, heterogenous, and familiar with mindfulness practice, with no control group. In addition, there was no measurement of how well participants engaged in the mindfulness task; therefore, it is possible the changes to state anxiety, affect, and state and trait mindfulness may have been attributable to other processes, such as “resting” in a virtual nature environment or distraction, irrespective of the mindfulness instructions. We sought participants’ perspectives on whether the Place app supports mindfulness practice, with responses indicating that the app was perceived to have this potential benefit. However, it is important to contextualize these findings; participants were not recruited based on expertise in mindfulness practice and varied in mindfulness experience. They could only reflect on their own experiences and use of the Place app in the given context. Furthermore, the qualitative interviews were semistructured, and the questions asked are likely to have influenced the data generated, while more open-ended prompts may allow themes to emerge more organically. Similarly, we asked participants to speculate about how they might use Place in future, generating some promising directions; however, these need to be validated via observation of app use within real-world contexts. Our participants’ perspectives provide helpful avenues for further investigation, and further research is essential to form any conclusions.

Future directions include assessment of the VR app in more ecologically valid settings, over a more prolonged course of mindfulness practice, and with a broader and more diverse sample, reflecting potential end users. Future research should also investigate how to increase the likelihood that participants will engage with the app: this might include enhancing the design of the VR experience. While this study provided some indications of potential future uses for the Place app, investigating end users’ engagement and interactions with the app in real-world contexts is an important next step. As noted, Place was designed as an experiential learning and on-the-spot self-management tool [41]. However, short-term and stand-alone mindfulness practices have been associated with altered neural activity as well as “downstream,” longer-term improvements [4,15,16,42], which may indicate generalization of skills in accordance with “offline training” [41]. This study used a one-off experience of the app in the laboratory context, and as such further research, supported by tailored measurement (eg, immediate vs generalized benefits) is needed to better understand the contexts in which the app may be beneficial.

Further investigation is needed to assess whether the VE is facilitating mindfulness or potentially other processes, such as distraction or relaxation. In addition, one possible use generated by participants in this study is VR mindfulness as a preventive measure. Furthermore, this study provided some preliminary signals of unique benefits of the app for different clinical groups, which warrant investigation within future research. A further unanswered question requiring confirmation is whether VR mindfulness confers additional benefits to traditional mindfulness practices for clinical groups. Promising avenues for future research therefore include rigorous clinical trials comparing outcomes for different clinical groups as well as comparing VR mindfulness with other mindfulness practices, evaluating the use of the app for prevention in both research and real-world contexts, assessing whether results can be generalized to VR mindfulness practices other than Place, and integration with other developing digital technologies, such as ecological momentary assessment and intervention.

### Conclusions

This study provides preliminary empirical support for VR-supported mindfulness having potential utility in those with mood or anxiety disorders. Evidence of proximal effects and perceived benefits support the potential for VR apps having a role in supporting mindfulness practice. While based on a single app, this study generated some evidentiary signals relating to potential future uses of VR mindfulness within clinical populations.

## Abbreviations

BD: bipolar disorder
FFMQ-SF: Five Facet Mindfulness Questionnaire Short Form
MBI: mindfulness-based intervention
MDD: major depressive disorder
PANAS: Positive and Negative Affect Schedule
TMS: Toronto Mindfulness Scale
VE: virtual environment
VR: virtual reality
